# Potential of Soft-Shell Rugby Headgear to Mitigate Linear and Rotational Peak Accelerations

**DOI:** 10.1007/s10439-022-02912-5

**Published:** 2022-01-20

**Authors:** Danyon Stitt, Natalia Kabaliuk, Keith Alexander, Nick Draper

**Affiliations:** 1 Department of Mechanical Engineering, University of Canterbury, Private Bag 4800, Christchurch, 8140 New Zealand; 2 School of Health Sciences, University of Canterbury, Christchurch, New Zealand

**Keywords:** Linear acceleration, Rotational acceleration, Rugby, Concussion, Headgear, Impact testing

## Abstract

Rugby union is a popular sport played across the world. The physical contact inherent in the game means that players are at increased risk of concussive injury. In 2019, World Rugby created a new category of permitted headgear under Law 4 as a medical device. This established a pathway for headgear designed to reduce peak accelerations to be worn in matches. Investigations of the potential of soft-shelled protective headgear to reduce head impact accelerations have been mostly limited to the analysis of linear kinematics. However rotational head impact accelerations have long been implicated as far more injurious. The aim of this study, therefore, was to assess the linear and rotational acceleration reduction brought about by soft-shelled rugby headgear. A Hybrid III headform and neck were dropped onto a modular elastomer programmer impact surface, impacting at four different velocities (1.7–3.4 m/s) in five different impact orientations. Impact surface angles were 0°, 30°, and 45°. Peak linear and rotational accelerations, PLA and PRA respectively, were recorded. All headgear significantly reduced PLAs and PRAs when compared to a no headgear scenario. The new generation, headgear reduced all measures significantly more than the older generation of headgear. Impact locations offset from the center of mass of the headform resulted in the highest PRAs measured. As the impact surface angle increased, both PLAs and PRAs decreased. The study demonstrated that headgear tested lowered PLAs by up to 50%, and PRAs by up to 60% compared to the bare headform. Our data suggest that new generation headgear could make a difference on the field in reducing injurious impact accelerations in a collision.

## Introduction

Rugby union is a popular sport played by nearly 9 million people across 121 countries worldwide.^53^ Due to the nature of the game, rugby players are at a heightened risk of experiencing injurious head impacts compared to the average person. These injuries are frequent, with several studies finding rates of concussion ranging from 0.4 to 46/1000 player hours,^13,23,32,36,42,57,58^ making it one of the most common injuries in the sport.^17,24,31,37^ This is not surprising as several studies investigating such injuries found players receive, on average, 14–52 significant (above 10 g peak linear acceleration) impacts to the head per game.^27,29,30^ Impacts over 10 g not resulting in acute concussion symptoms, have been labelled sub-concussive impacts.^3^ It has been suggested that combinations of concussive and sub-concussive head impacts, may result in long-term conditions such as chronic traumatic encephalopathy,^18^ cognitive impairment^21^ and depression.^22^ When the head experiences impact accelerations, the difference in density causes parts of the brain to accelerate at different rates, causing stresses and strains to develop in the brain tissue.^20^ The brain can handle some deformation, however once a certain threshold is surpassed, trauma occurs, giving rise to the short term symptoms of concussion such as loss of balance and memory.^20^

Several research articles investigating the acceleration reduction offered by rugby headgear have been published,^12,16,33,39^ however, almost all exclusively focus on linear kinematics. Experiments investigating animal response to acceleration loading demonstrated that rotational acceleration was associated with greater diffuse axonal injury and greater shear stresses within the brain tissue.^2,11,19,25,34,44,45,48,60^ Holbourn used shear strain patterns in 2D gel models to claim that translational acceleration is not injurious, but rotational accelerations are.^25^ Genneralli *et al*. found squirrel monkeys became concussed only when subjected to purely rotational motion, but not purely translational motion.^19^ Denny-Brown and Russell found transient loss of consciousness was induced only when the head was free to rotate but not when the same forces were applied to a fixed head.^11^ A review of non-human primate studies found that loss of consciousness and coma were rarely obtained with impacts causing primarily linear accelerations, but occurred frequently at much lower impact thresholds when the head was free to rotate. The same was found for diffuse axonal injury.^8^

Despite literature suggesting that rotational kinematics pose a greater injury risk than their linear counterpart, it is not well understood, in the context of the human brain, whether linear or rotational acceleration causes greater structural damage. The comparability to human data for these is argued within the literature, as there are structural, composition, and size differences which complicate the comparison.^8^ Additionally, in real life scenarios, head impacts rarely cause purely linear or rotational motion. There is also debate about which of the impact kinematics has the most influence on concussive injury severity and the associated recovery (return to play) times.^40,51,52^ What is agreed, however, is that a reduction of all accelerations seen in an impact is likely to reduce the risk of sustaining a concussive injury.

Rugby headgear has been shown to significantly reduce the peak linear acceleration (PLA) seen in laboratory impacts.^12,16,33,39^ Apart from Ganly *et al*., there have been no investigations into the peak rotational acceleration (PRA) reduction offered by rugby headgear. Unfortunately, the methods employed by Ganly *et al*. to generate rotational accelerations were detailed minimally.^16^ The World Rugby impact testing standard requires headgear be fitted to a metal headform conforming to EN960 and dropped onto a flat impact surface, also made of steel, from a height corresponding to 13.8 J impact energy. Five impact locations are chosen (top, left and right temple regions and side regions) with each subject to one impact each. Headgear meets the criteria for attenuation if the PLA is greater than 200 g. Additionally, headgear has strict thickness and density limits imposed, limiting both to 10 ± 2 mm and 45 kg/m^3^ respectively. In the recently developed law 4 trial assessment, the attenuation limit and density limit have been removed, however both only require linear acceleration testing to be carried out.^54,55^ It should be noted that there are several other conditions that must be met for headgear to be approved by World Rugby including retention system strength and effectiveness, and visual field obstruction conditions. Since these do not pertain to impact attenuation performance, these will not be reviewed in depth.

Given there is sufficient literature suggesting that PRA is far more concerning in terms of the injury potential, it makes sense to assess the headgear for both linear and rotational kinematics mitigation. Several studies have already investigated rotational performance of head protection devices for activities where head injuries are prevalent. Most have devised their own methods to generate rotational kinematics in a laboratory setting. Aare and Halldin investigated the PLA and PRA reduction of motorcycle helmets using an instrumented Hybrid III (HIII) dummy head.^1^ The head alone was dropped without constraint onto a moving impact surface in three different orientations allowing an “effective” impact velocity of up to 14 m/s. McIntosh *et al*. used a similar method for testing bicycle helmets, however, the authors used a HIII head and neck attached to a drop assembly. This was dropped onto a horizontally moving impact surface from a single height of 1.5 m.^38^ Bland *et al*. also investigated bicycle helmets using a free-fall simulation drop test rig. The authors used a HIII and a NOCSAE headform with a neck attached, which was in turn attached to a 16kg torso mass. This assembly was dropped onto an angled impact surface (45°) at an impact velocity of 6m/s. Head and neck orientations were selected based on impact locations common in cyclist accidents.^5^ Similarly, Bottlang *et al*. used a HIII headform and neck to investigate PRA mitigation of bicycle helmets. The assembly was dropped onto an impact surface angled at 45°.^7^ Bliven *et al*. similarly used drop testing onto an impact surface angled at 30°, 45°, and 60° to investigate PRA reduction of bicycle helmets.^6^

World Rugby have designed the approval standards for headgear in a way that limits overprotection of players. World Rugby states that the specifications for padded clothing and headgear are intended to encourage players to protect themselves rather than provide equipment that would materially provide injury protection. World Rugby also explicitly states that headgear approved by world rugby is not intended to protect against any form of mild traumatic brain injury or skull fractures. Despite this, World Rugby approved headgear is often purchased by parents, for instance, to help protect their children from head injuries.

This study, therefore, aimed to assess various common off-the-shelve soft-shelled rugby headgear for both linear and rotational acceleration reduction using a drop testing method inspired by the body of literature from other high concussion rate sports and the standards set by World Rugby.

## Materials and Methods

### Headgear Choice

**Figure 1:**
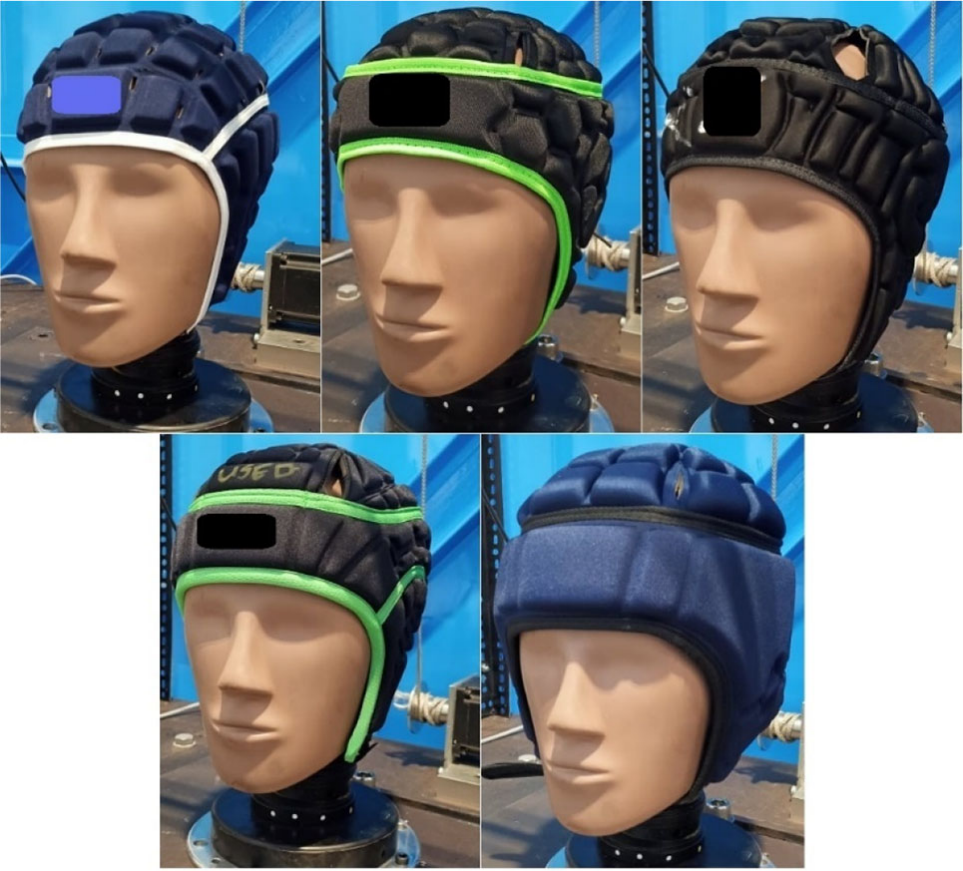
Headgear 1-5 assessed in this study from top left to bottom right: CCC—1, Gilbert—2, 2nd skull—3, Npro—4, Gamebreaker pro—5.

Headgear units used in this study were: CCC Reinforcer, Gilbert Falcon 200, 2nd Skull, N-Pro, and Gamebreaker Pro (hereon referred to as headgear 1–5 respectively), all of which were in the medium size as it best fit the circumference measurement of the headform across all headgear tested (Fig. 1). These were chosen based on popularity within the game (as observed by the authors across all levels of play) and World Rugby approval. Headgear 5 was the only headgear not approved by World Rugby at the time of the investigation but was included as linear testing of the headgear showed it could reduce the PLA and HIC significantly more than all other headgear tested.^12^ It should be noted that World Rugby approved headgear (prior to the law 4 trial approval process) is not designed to mitigate risks of brain injury or skull fracture. Approved headgear does, however, serve as an appropriate baseline for comparison to newer models of headgear with potential to lower concussion risk.

Headgear 1 is made using lightweight polyurethane foam (≤ 45 kg/m^3^) arranged into rectangular cells around the headgear. Headgear 2 is made of lightweight (≤ 45 kg/m^3^) polyethylene foam formed into cells replicating the shape of the logo. Headgear 3 uses a light weight (≤ 45 kg/m^3^) ethylene vinyl acetate (EVA) foam arranged in honeycomb shaped cells. Headgear 4 uses a thicker, higher density (≥ 45 kg/m^3^) open cell polyurethane foam in square cells of varied size.^16^ Headgear 5 uses EVA foam and a layer of impact absorbing foam developed by D3O^®^ (≥ 45 kg/m^3^).^15^ Headgears 1, 4 and 5 use foams that are viscoelastic and open celled compared to headgears 2 and 3; which use closed cell foams. Headgear 5 was the thickest unit (15–20 mm max thickness), compared to headgear 4 (12–13 mm max thickness) and headgear 1–3 (8–10 mm max thickness). Measurements were taken from the forehead, rear boss and side locations for all headgear, as these were the most easily accessible areas. All headgear fit tightly on the headform with no slippage ensuring a consistent impact region throughout testing. This was crucial as the reliability of rotational data can be compromised if headgear is not properly coupled to the headform. All headgear was new and in unused condition.

### Study Design

**Figure 2:**
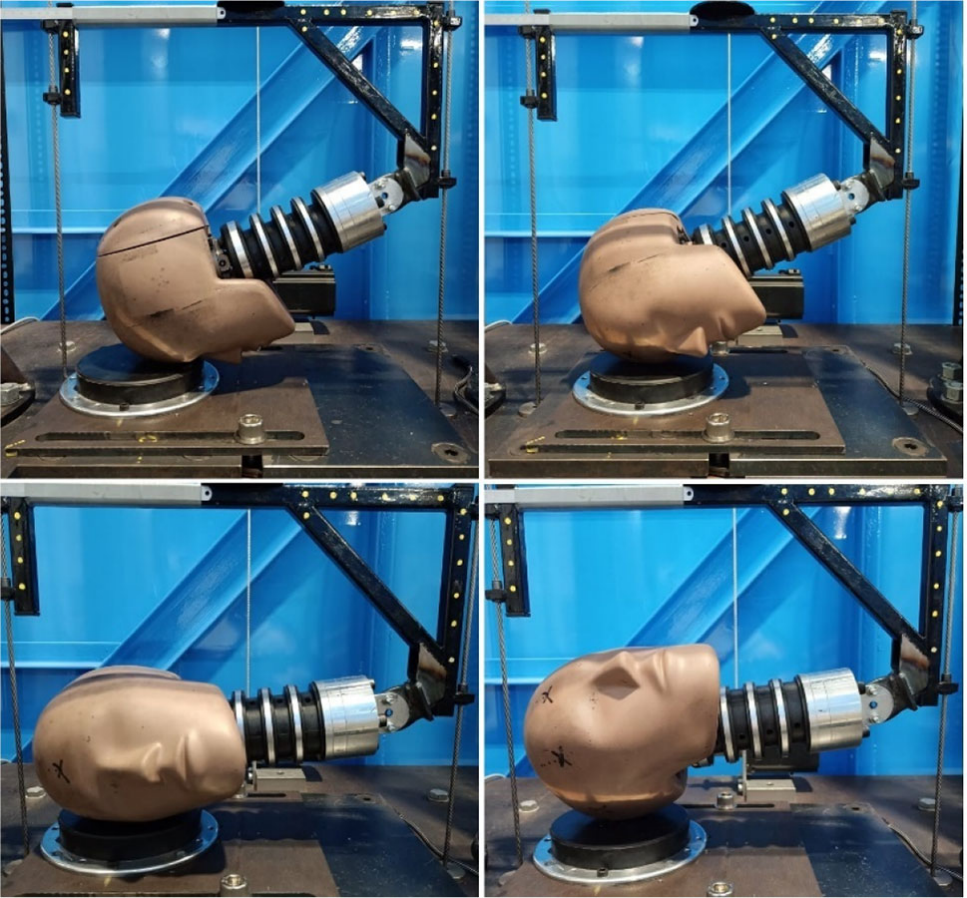
Impact locations for the flat MEP pad testing from top left to bottom right: forehead, Front boss, Side, RR boss.

**Figure 3:**
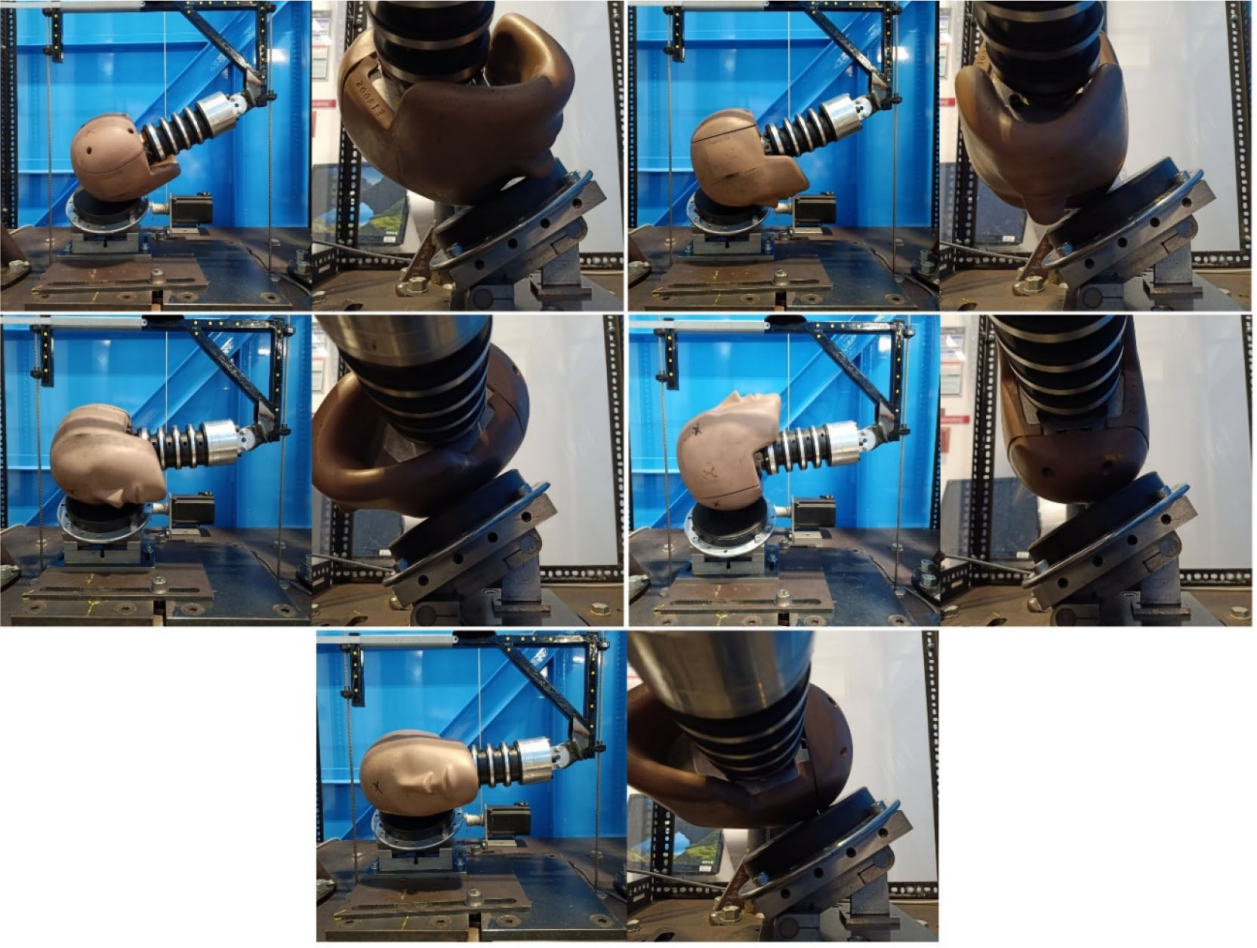
Impact locations and MEP pad contact area for the 30 and 45° impact surface. From top right to bottom: forehead, front boss, Side, Rear, RR boss, and SR boss. The angle shown here is 30°.

Headgear impact testing was carried out using a twin wire guided drop test rig with a 1-inch MEP pad as the impact surface. Tests were performed with the headform and neck from a 50th percentile male Hybrid III dummy. The neck was torqued to 1.4 Nm as required by Humanetics, and was checked after every hour of testing. Additionally, reference impacts were carried out on each day of testing using a bare headform, with the PLA and PRA recorded to ensure there were no consistent changes following prolonged testing. Impacts were carried out with the impact surface at 0°, 30°, and 45° relative to the surface underneath (Figs. 2 and 3). Impact locations onto the 0° (flat) impact surface are shown in Fig. 2. These locations were: forehead, front boss, side, and rear boss. Rear impact locations were excluded as only headgear 5 provides padding at the back. The top of the head was also excluded as field studies have shown this location to be the least commonly impacted region during gameplay.^27,28,30^

A fifth impact location was introduced for the 30° and 45° impacts (Fig. 3). This extra impact region was termed side rear boss (SR boss), chosen to induce a large amount of rotation of the headform. As such the standard rear boss location is referred to hereon as rear, rear boss (RR boss). All impact locations were to padded areas of the headgear however, motion of the head during impact may have resulted in some part of the impact force being applied to a seam, or area of low padding. The effects of this were assumed to be negligible, as the initial impact site was always on the padded areas, and therefore the effects were not investigated.

Four drop heights were chosen from the World Rugby law 4 trial assessment (150, 300, 450, and 600 mm) (Table 1).^55^ Heights above 600 mm were avoided to reduce the chance of damage to the head and neck. Impacts were repeated 5 times each with 60 seconds between each consecutive impact. Since it is not known how much of the total falling mass is involved in the acceleration peak, impact energies in Table 1 were determined for the head and neck only (5.6 kg), and the total falling mass of 6.8 kg including the drop frame. For easier comparability to other studies, results were reported in terms of the impact velocity.

**Table 1:** Impact parameters.

Drop height (mm)	Impact velocity (m/s)	Head/neck impact energy (J)	Whole system impact energy (J)
150	1.7	8.2	9.8
300	2.4	16.1	19.6
450	3.0	25.2	30.6
600	3.4	32.4	39.3

It should be noted that this study did not intend to exactly recreate the previous literature on bicycle helmets, nor did it intend to recreate the World Rugby standards. The present study used them as a base from which to extend the investigation of headgear behaviour. This study did not test headgear for World Rugby approval but assessed and compared the impact attenuation behaviour of selected headgear.

### Data Acquisition and Statistical Analysis

The headform housed four tri-axial accelerometers (Analog Devices ADXL377, range: ± 200 g, sensitivity: 6.5 mV/g) for a total of 12 sensing axes. Accelerometers were configured in a standard "Nine Accelerometer Package" (NAP) array^47^ with the three redundant sensing axes configured radially along each primary axis. Accelerometer data was processed to find linear and rotational acceleration at the centre of mass of the headform, according to the standard NAP algorithm.^47^ Once a kinematic solution was found, results were projected back to the location of each accelerometer and cross-checked with their actual reading, thereby allowing identification of capture errors such as deformation.

Severity of the impacts was compared using the PLA and PRA. In addition to these, the Head Injury criterion (HIC) and Rotational Injury Criterion (RIC) scores were used to help quantify shape differences in the acceleration traces. HIC was developed by the National Highway Traffic Safety Administration and focuses the severity index on the part of the acceleration trace most relevant to the risk of serious brain and skull trauma.^61^ Specifically, HIC_15_ was used, where t_1_ and t_2_ were 15 ms apart. RIC (developed by Kimpara *et al*^26^) focuses the severity index on the part of the rotational acceleration trace most relevant to the risk of brain injury. This is taken over a 36ms time span which, similarly to HIC, leads to the maximum possible value of RIC. It is still debated whether these metrics reliably predict concussive injury likelihood. They do, however, quantify the differences in the shapes of the acceleration traces, which is information peak values alone do not convey. Both HIC and RIC were calculated as shown in Eq. 1 and 2:

1 $$HIC = \left( {t_{2} - t_{1} } \right)\left[ {\frac{1}{{t_{2} - t_{1} }}\mathop \smallint \limits_{{t_{1} }}^{{t_{2} }} a\left( t \right)dt} \right]^{2.5}$$

2 $$RIC = \left( {t_{2} - t_{1} } \right)\left[ {\frac{1}{{\left( {t_{2} - t_{1} } \right)}} \mathop \smallint \limits_{{t_{1} }}^{{t_{2} }} \alpha \left( t \right)dt} \right]^{2.5}$$

With ‘*a*’ denoting the linear accelerations and ‘*α*’ denoting the rotational accelerations, *t*_1_ and *t*_2_ are the start and finish times of the respective injury metric.

To analyse overall headgear behaviour, a composite average was taken as the average peak acceleration across all orientations for each of the five impact repeats (Eq. 3). This was done for each height and headgear, and was carried out for the HIC and RIC values. Distributions, descriptive statistics, and mixed design ANOVAs were calculated using SPSS (Version 25, IBM SPSS Statistics Inc., Chicago, IL, USA). The data was assessed for violations of the assumptions of normality of distribution using the Shapiro-Wilk test, with results showing a Gaussian distribution. *p* ≤ 0.05 was set for accepting statistical significance.

3 $${\text{Comp}}\;{\text{PLA}}\;{\text{drop}}\;1 = \frac{{{\text{drop}}\;1\left( {{\text{forehead}}\;{\text{PLA}} + {\text{front boss}}\;{\text{PLA}} + {\text{side}}\;{\text{PLA}} + {\text{RR}}\;{\text{boss}}\;{\text{PLA}} + {\text{SR}}\;{\text{boss}}\;{\text{PLA}}} \right)}}{5}$$

## Results

### Composite Peak Accelerations

**Figure 4:**
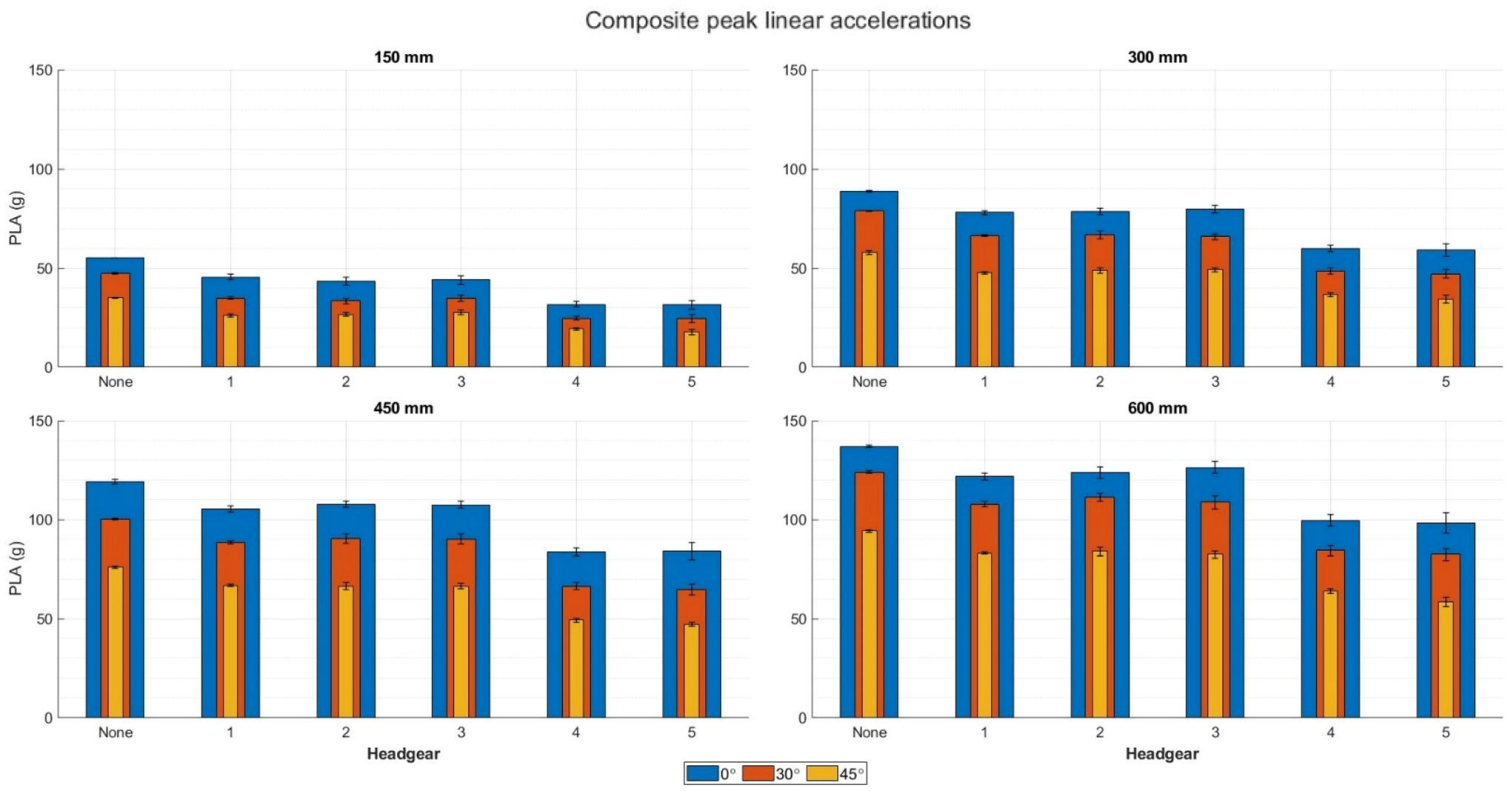
Composite peak linear accelerations of each headgear compared to no headgear (labelled: none). The blue bars show the results from a flat impact surface, the orange show the 30° impact surface, and the yellow show the 45° impact surface.

All headgear significantly reduced the composite PLAs compared to a no headgear scenario at 0° impact surface angle at all four impact velocities (*p* < 0.05) (Fig. 4). No significant difference was seen in PLA reduction between headgears 1–3. Headgear 4 and 5 reduced PLA’s significantly more than headgear 1–3 (*p* < 0.05), but did not significantly differ from each other at any impact velocity.

Similarly, at 30° impact surface angle, all headgear significantly reduced the PLA compared to no headgear (*p* < 0.05) (Fig. 4). No significant difference in PLA was seen between headgears 1–3 at any impact velocity except 3.4 m/s where headgear 1 reduced the PLA significantly more than headgear 2 (*p* = 0.024). Headgear 4 and 5 reduced PLA significantly more than headgear 1–3 (*p* < 0.05), however, neither reduced PLA more than the other.

When dropped onto a 45° impact surface, all headgear significantly reduced PLAs at all four impact velocities (*p* < 0.05). At 1.7 m/s, both headgears 1 and 2 reduced the PLA significantly more than headgear 3 (*p* = 0.018), however at all other impact velocities the difference did not reach significance. At all impact velocities, headgear 5 reduced the PLA’s significantly more than headgear 4 (*p* < 0.05). The difference between the two increased as the impact velocities increased (from 1.7 g at 1.7 m/s to 5.8 g at 3.4 m/s).

**Figure 5:**
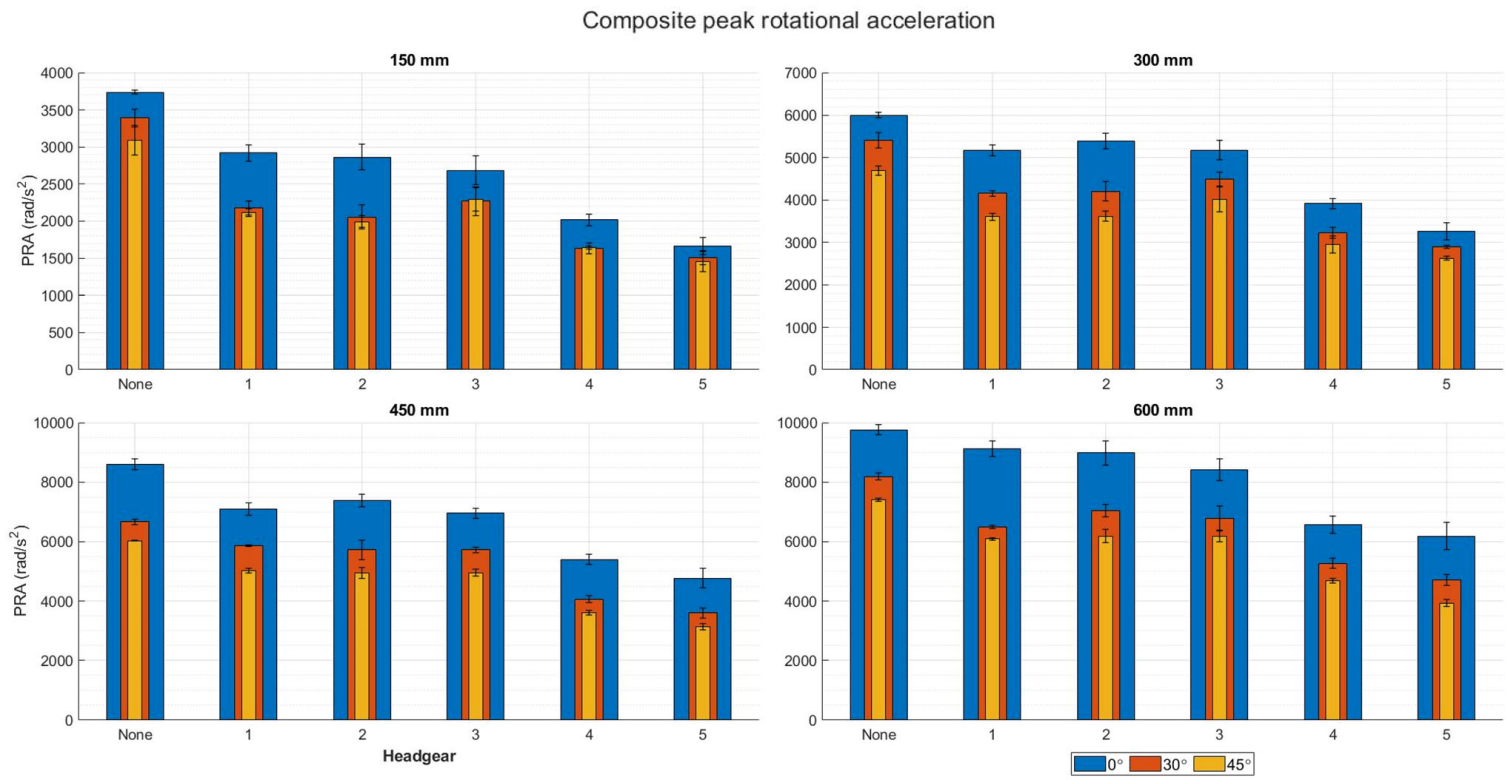
Composite peak linear accelerations of each headgear compared to no headgear (labelled: none). The blue bars show the results from a flat impact surface, the orange show the 30° impact surface, and the yellow show the 45° impact surface.

When dropped onto a 0° impact surface, all headgear significantly reduced the PRA at all impact velocities (*p* < 0.05) (Fig. 5). At 1.7 and 3.4 m/s, headgear 3 reduced PRA significantly more than headgear 1 and 2 (*p* < 0.05). At 2.4 and 3.0 m/s, headgear 1 and 3 reduced PRAs significantly more than headgear 2, however, this difference barely reached significance in both cases (*p* = 0.045). Headgear 5 reduced PRAs significantly more than headgear 4 at 1.7, 2.4, and 3.0 m/s (*p* < 0.05), however neither 4 nor 5 produced significantly different PRA reduction at 3.4 m/s.

All headgear significantly reduced PRA’s at all impact velocities compared to no headgear on the 30° impact surface (*p* < 0.05). At 1.7 m/s, headgear 2 reduced the PRA’s significantly more than headgear 3 (*p* < 0.05). At 2.4 m/s, headgear 1 and 2 reduced the PRA’s significantly more than headgear 3 (*p* < 0.05). Headgear 1 and 2 did not significantly differ by PRA reduction. At 3.0 and 3.4 m/s, headgear1–3 did not significantly differ by PRA reduction. Headgear 5 reduced the PRA’s significantly more than headgear 4 at all impact velocities above 1.7 m/s (*p* < 0.05).

At 45° impact surface, all headgear significantly reduced PRA’s compared to no headgear across all impact velocities (*p* < 0.05). Headgear 5 reduced the PRA’s significantly more than headgear 4 at all impact velocities (*p* < 0.05). At 1.7 and 2.4 m/s, headgear 1 and 2 reduced the PRA’s significantly more than headgear 3 (*p* < 0.05). Headgear 1 and 2 did not differ significantly at these impact velocities. At 3.0 and 3.4 m/s, headgear 1–3 did not differ significantly compared to each other.

Overall, as the impact surface angle increased from flat, the PLA and PRA decreased significantly (*p* < 0.05). There is an exception to this case at 1.7 m/s, where both headgear 3 and 4 showed higher PRAs for 45° impact surface than the 30° impact surface. This was not repeated as the impact velocity increased. Headgear 4 and 5 reduced the PLA and PRA significantly more than headgears 1–3 across all impact velocities and impact surface angles (p < 0.05).

### Injury Criteria

All headgear significantly reduced the HIC and RIC values compared to no headgear (p < 0.05) (Figs. 9 and 10). These results are not discussed in depth as the trends in HIC and RIC reduction are similar to those for PLA and PRA. Headgear 4 and 5 reduced the HIC and RIC significantly more than headgears 1–3 at all impact velocities (*p* < 0.05). Headgears 1–3 did not consistently significantly differ from each other across the 4 IVs for both HIC and RIC. Like the PLA and PRA measures, the HIC and RIC values decreased as the impact surface angle increased. There were a couple of exceptions to this at 1.7 m/s for RIC, where headgear 1–3 displayed higher values for the 45° impact surface than the 30° impact surface.

### Directional Results

**Figure 6:**
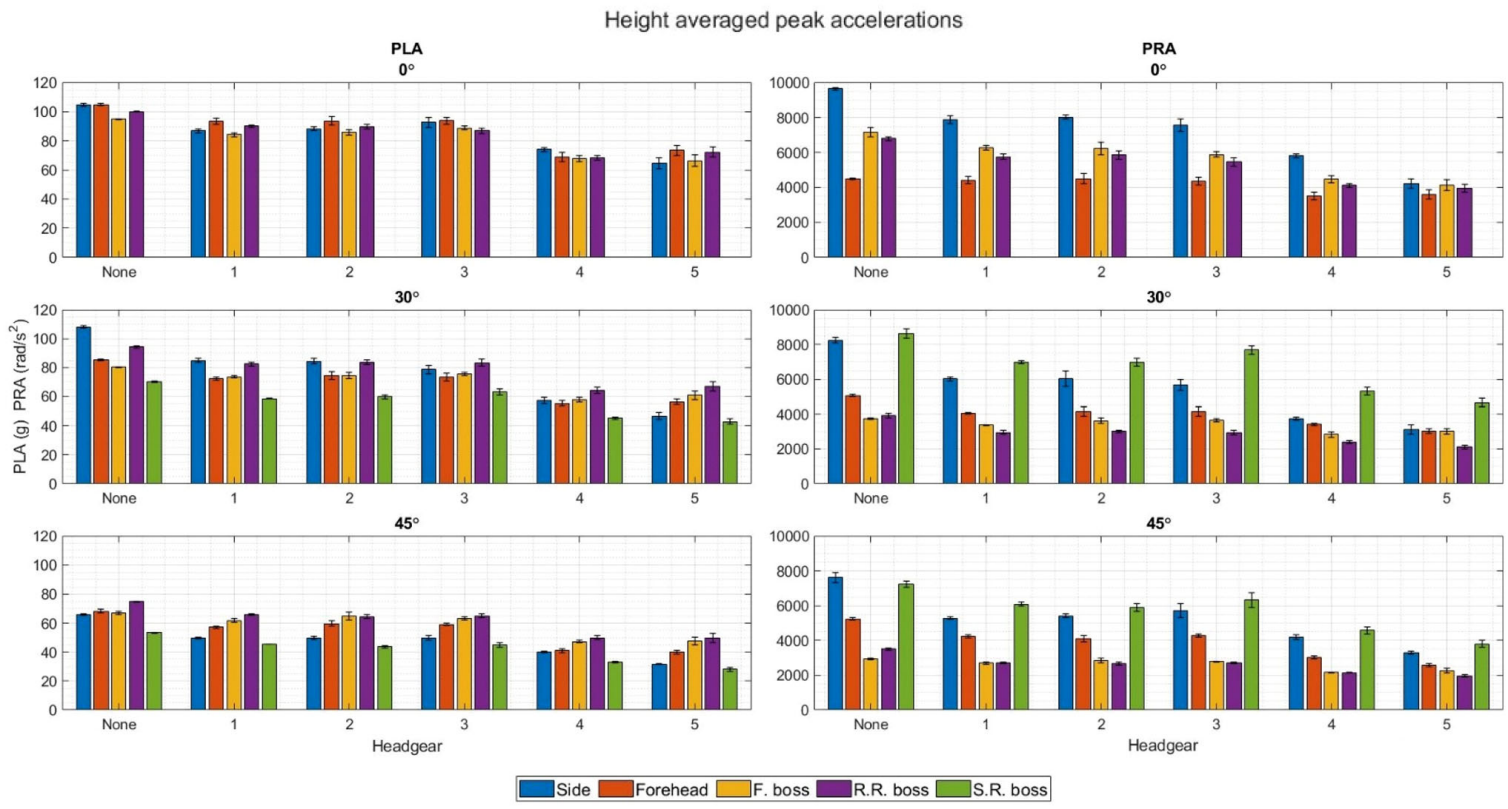
Height averaged PLAs and PRAs for each headgear, impact location, and impact surface angle.

The impact location specific PLAs and PRAs behaved the same between the different impact locations across the four impact velocities for each impact surface angle. Therefore, an average was taken, combining the four impact velocities into one (Fig. 6). Higher PLAs did not necessarily correspond to higher PRAs in each impact location. An example of this can be seen in Fig. 4 where the highest PLA on a 30º impact surface is produced in the RR Boss position (except for no headgear and CCC), however this impact location displays some of the lowest PRAs of all impact locations for the same impact surface angle. The same was observed for the 45° impact surface where the two impact locations producing the highest PRA’s produced the lowest PLAs compared to the other impact locations.

Side and SR boss positions consistently displayed the highest PRAs across all headgear, impact velocities, and impact surface angles. Headgear 4 and 5 displayed the highest PLA and PRA reduction percentages across all impact locations, impact velocities, and impact surface angles. Front boss impact locations displayed the lowest PLA and PRA reduction when the angle of the impact surface was increased from flat. No consistent significant difference in PLA or PRA reduction was seen between 30° and 45º impact angles for any of the headgear. Headgear 4 and 5 showed much lower differences between the highest and lowest PRAs than the other headgear.

### Acceleration Reductions

Figure 11 shows the percentage reduction of the PLAs and PRAs relative to no headgear for each impact location, averaged across all four impact velocities. The side impact position displayed the highest PLA and PRA reduction across all surface angles except for headgear 4 PLA on a flat impact surface. The trends in PLA and PRA percentage reduction across the different impact locations were similar for each headgear. The side displayed the highest percentage reduction, followed by the SR boss, RR boss, and the forehead, with the front boss location consistently showing the lowest PLA and PRA reduction. Headgear 4 and 5 visibly outperformed the other headgear in terms of percentage reduction.

**Figure 7:**
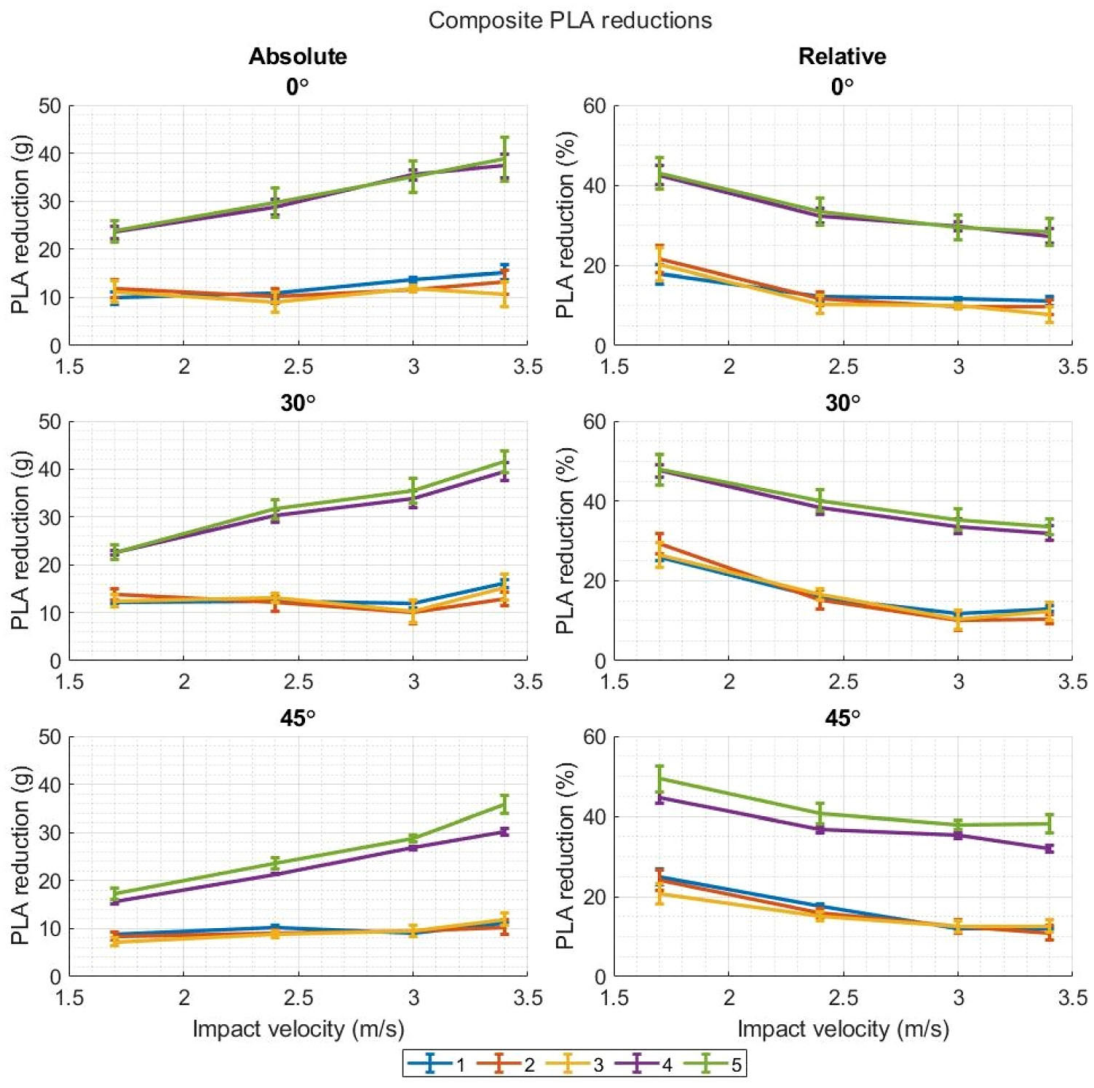
Absolute and relative reductions of the composite PLAs as the impact velocity/drop height increases. Results are shown for each impact surface angle.

**Figure 8:**
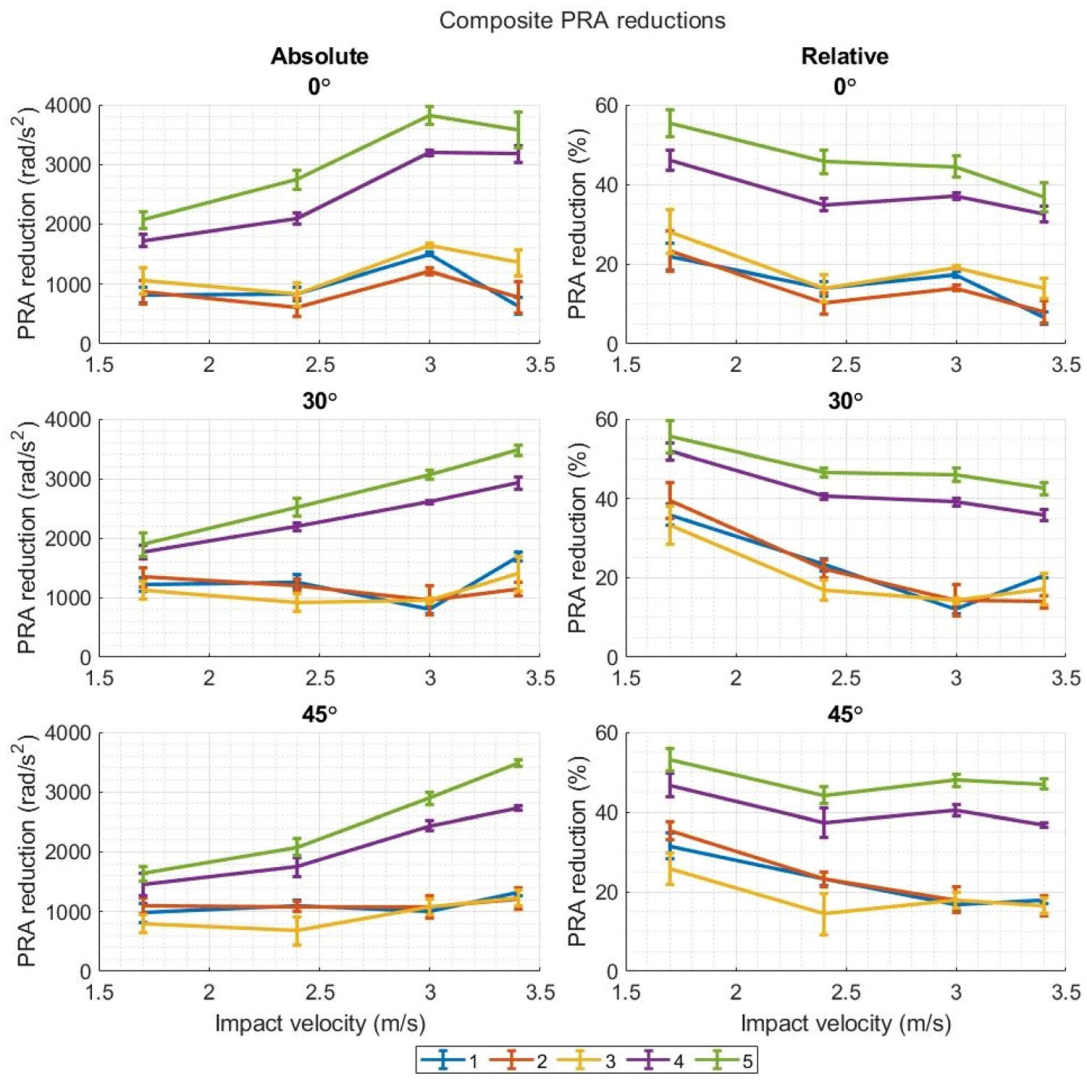
Absolute and relative reductions of the composite PRAs as the impact velocity/drop height increases. Results are shown for each impact surface angl.e

**Figure 9:**
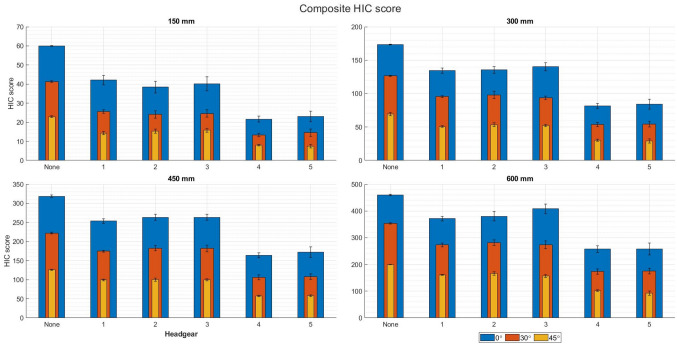
Composite HIC score of each headgear compared to no headgear (labelled: none). The blue bars show the results from a flat impact surface, the orange show the 30° impact surface, and the yellow show the 45° impact surface.

**Figure 10:**
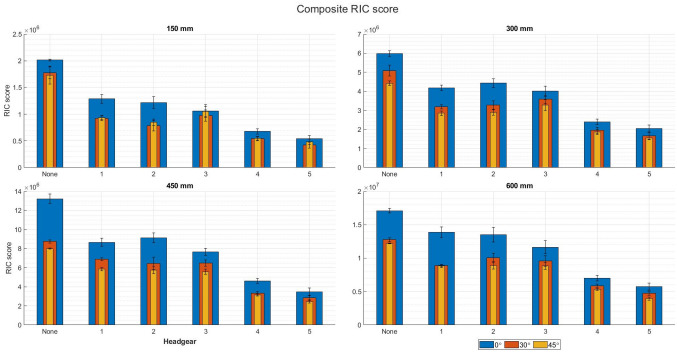
Composite RIC score of each headgear compared to no headgear (labelled: none). The blue bars show the results from a flat impact surface, the orange show the 30° impact surface, and the yellow show the 45° impact surface.

**Figure 11:**
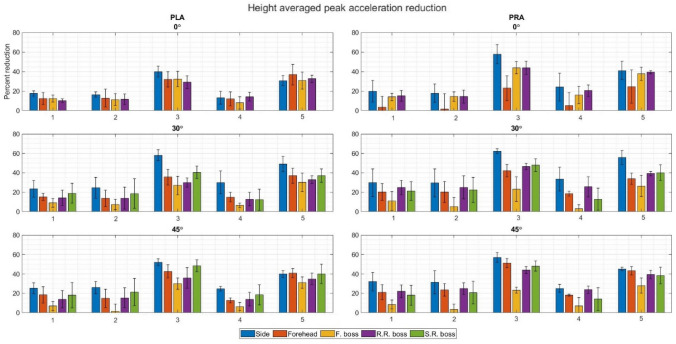
Height averaged peak acceleration reductions relative to no headgear for each impact location and impact surface angle.

**Figure 12:**
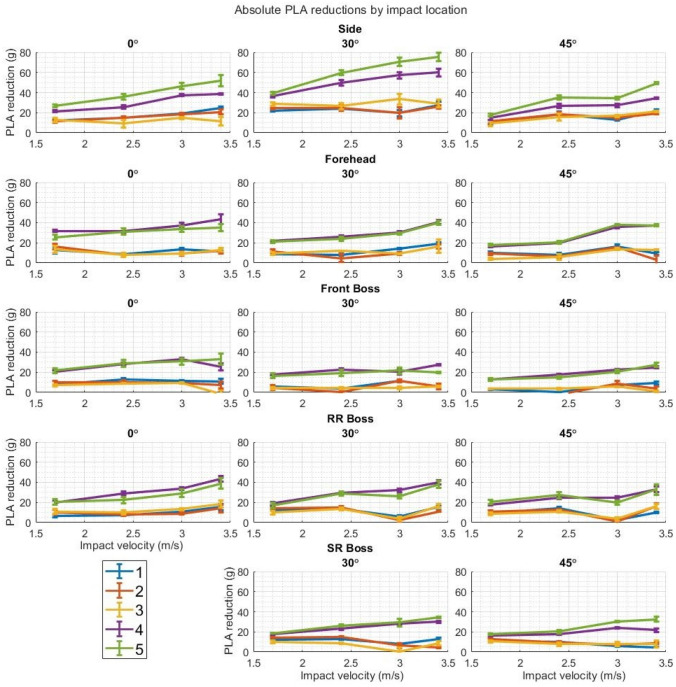
Absolute PLA reductions separated by impact location

**Figure 13:**
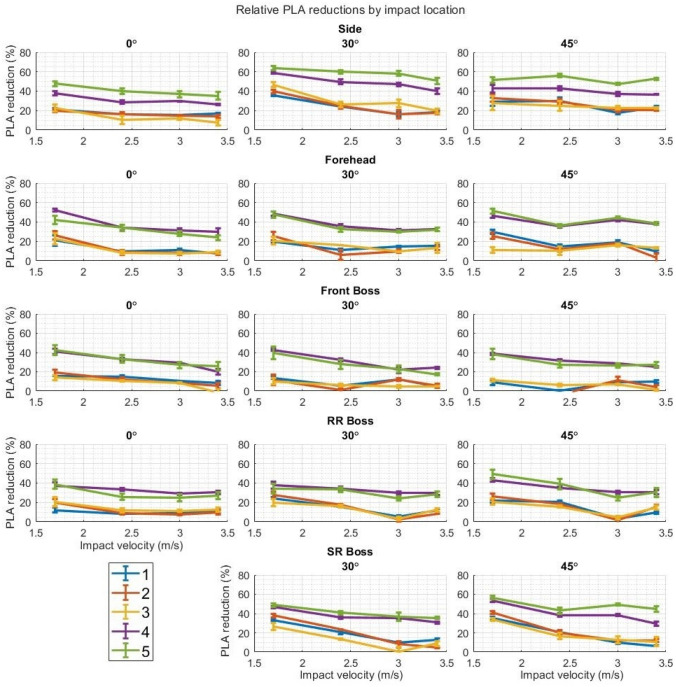
Relative PLA reductions separated by impact locations.

**Figure 14:**
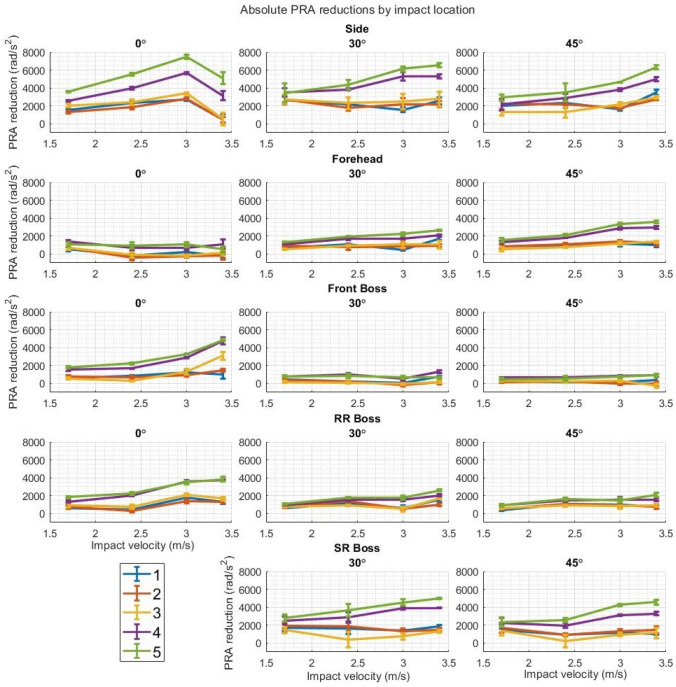
Absolute PRA reductions separated by impact location.

**Figure 15:**
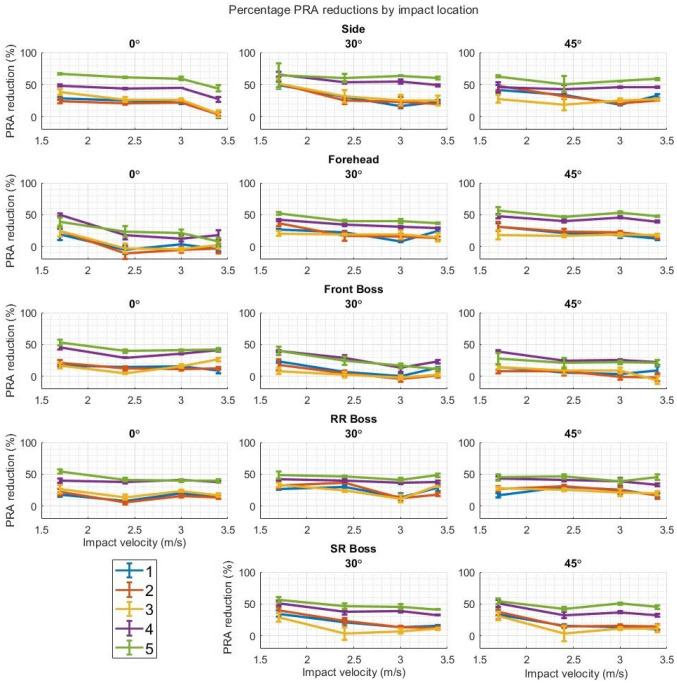
Relative PRA reductions separated by impact location.

Figures 7 and 8 (and Figs. 12, 13, 14, and 15) show the absolute reduction and percentage (relative) reduction of composite PLA and PRA of each headgear as the impact velocity increases. There was a clear divide between headgears 1–3 and headgear 4 and 5, with the latter showing much higher PLA and PRA reductions (both absolute and percentage) across nearly all impact velocities, impact locations, and impact surface angles. Headgears 1–3 reduced the PLA and PRA to effectively the same extent as each other. There were some cases where one may have reduced these measures more than the other headgear, however, this was not consistent across the entire range of data. As the impact velocity increased, there was little increase in the absolute PLA and PRA reduction displayed by headgears 1–3 across any impact location. This reduction remained relatively consistent as the drop height increased. The absolute PLA and PRA reduction displayed by headgear 4 and 5 increased as the impact velocity increased, except for the front boss PLA and PRA as the impact surface angle increased from flat. However, the percentage reduction in PLA and PRA showed a substantial decrease for all headgear as the impact velocities increased across all impact locations and impact surface angles.

## Discussion

### Overview

All headgear significantly reduced the composite peak accelerations seen in an impact compared to no headgear. For the PLAs, this is not surprising as nearly all published literature on this subject agrees.^12,16,33,39^ This is expected as the presence of a foam padding extends the time of total deceleration, thereby decreasing the peak acceleration. Headgear was visibly divided into two separate groups in terms of acceleration reductions. These two groups were comprised of headgears 1–3 and headgear 4 and 5, with the latter consistently reducing the peak accelerations significantly more than the first group. This behaviour was observed across all impact locations and all impact velocities.

All headgear significantly reduced the HIC and RIC injury metrics compared to a no headgear scenario. The general behaviour observed with the HIC and RIC was consistent with that of the PLA and PRA. Both these metrics are based off the linear and rotational accelerations. The equations used to calculate these metrics focus on the part of the acceleration trace that results in the highest value of HIC and RIC. This almost always occurs about the peak acceleration. It makes sense therefore, that the HIC and RIC would largely follow the same behaviour as the peak accelerations.

### Foam Mechanics

Headgears 1–3 incorporate similar materials (≤ 45 kg/m^3^ foam,^9,59^ 8–10 mm max thickness), in similar cell structure arrangements around the headgear to provide impact attenuation. This is likely why all three were rarely significantly different from each other. Headgear 4 utilised a high density, viscoelastic, open cell polyurethane foam^16^ while headgear 5 used a layer of EVA foam^14^ and a layer of impact absorbing, viscoelastic foam developed by D3O^®^.^14^ The higher density, viscoelastic, open cell foams used in headgear 4 and 5 dissipated a much greater proportion of the impact energy than the lower density or closed cell foams used in headgear 1–3.

The mechanical behaviour of a foam depends on its structural properties, thickness, strain rate, and density.^35,41,46,56^ Closed-cell foams are made up of many tiny pockets of air trapped within cells made of the foam polymer. Energy is primarily absorbed through compression of the air pockets inside, and deformation of the cell walls giving the foams their ‘springy’ feel when compressed.^41,43,46^ Above certain strains, closed-cell foams undergo plastic deformation, destroying the cell structures and allowing air to escape, permanently damaging the foam.^49^ Open-cell foam cells are not fully closed off, allowing air to move through the material. Energy is absorbed through deformation of the polymer structure which forces air through the cellular structure. The viscous forces created by air moving through the foam also dissipate a significant amount of energy.^35,41,46^ Headgear 4 and 5 both exhibited a noticeable ‘memory foam’ effect when deformed. This is likely due to the glass transition temperature of the foams being near that of the laboratory temperature, allowing significant deformation of the foam structure without permanent damage.^10^ This allows the foam to restore its original shape after some time.

Open cell foams are far less stiff than the equivalent density closed cell foams,^49^ therefore, much higher density open-cell foams can be used in ‘soft-shelled’ headgear than what is possible for closed cell foams. This increased foam density, and increased viscoelastic response of the open cell foams, likely accounts for most of the difference in impact attenuation behaviour between the two foam types. Unfortunately, there is not a great deal of literature on the impact attenuation behaviour of the flexible foams used in headgear as the density is varied. The authors of the present study, however, speculate there could be a specific desired density for each foam, at which, the impact attenuation is optimised, and following which, there is an excess of solid phase in the foam, reducing the attenuation performance.

### Note on Terminology

As the impact velocity increases, the percentage reduction of the PLA and PRA by the different headgear reduces across the board (Figs. 6 and 7). This result is reported commonly throughout the literature, and in previous work by the authors of the present study. However, this may be a misleading way of describing the observed behaviour. When reported in such a way, it seems the headgear “is less effective” as the impact velocity increases.^12^ This is not necessarily the case. Absolute reduction of PLAs and PRAs can increase as the impact velocity increases; however, the measured PLAs and PRAs from the impact increase at a greater rate than the reduction amount does, leading to the apparent attenuation decrease. This does not mean that percentage reductions are a worthless or disingenuous measure, as it is a valid way of normalising the results for comparison between headgear and impact velocity. It should however be known that, on its own, percentage reduction does not fully explain what is occurring.

Headgears 1–3 showed little to no increase in absolute PLA and PRA reduction as the impact velocity increased, whereas headgear 4 and 5 displayed significant increases in the absolute PLA and PRA reductions as the impact velocity increased. Headgear 2 and 3 use closed cell foams where, in impact loading, the viscoelastic behaviour of the polymer, and the rapid compression of air inside limits the amount of deformation the headgear can undergo before failure of the polymer structure. Thus, limiting the amount of impact energy absorbed through the headgear. Headgear 1 uses a low-density open-cell foam. Open cell foams are known to be much less stiff and strong than the closed cell foams, therefore it is believed the foam is bottoming out, limiting the impact energy absorbed. This behaviour was not seen in headgears 4 and 5 for the present test conditions, however it is hypothesized there is a point, as the impact velocity and energy increases further, where these foams no longer provide increasing acceleration reduction.

### Effect of Thickness

The performance differences between headgears 4 and 5 can likely be attributed to the difference in the thickness, as headgear 5 had a greater thickness than headgear 4. The increased thickness is likely the reason headgear 5 reduced the composite PRAs significantly more than headgear 4 across all impact velocities. Since none of the main impact absorbing layers of the foam have been tested on their own, it is not possible to draw conclusions on which of the foams from headgear 4 or 5 perform better in isolation.

### Impact Location Differences

For flat impacts, side impact locations produced the highest PRAs and some of the highest PLAs across all headgear. As this is also seen with a bare headform, it is most likely caused by the intrinsic properties of the headform and neck system. By design, side impacts do not elicit movement from the occipital condyle joint. Only forehead and rear impacts allow full inclusion of motion about this joint. Side impacts therefore exclude a fundamental mode of motion, leaving lateral neck bending as the primary mode of flexural motion. This increases the effective stiffness of the system, increasing accelerations.^4^

Similar to previous work with HIII dummies, the side and SR boss impact locations both created much higher PRAs than the other impact positions across the three impact surface angles.^62–64^ During both side and SR Boss impacts, the direction of the force does not travel through the headform centre of mass, rather it is offset. This offset is larger in SR Boss impacts than side impacts. Due to this offset, when the headform contacts the impact surface, a torsional rotation about the axis of the neck occurs. This is also why the SR Boss position records the lowest PLA values. A large amount of torsional rotation around the neck occurs. The headform and neck deflect and travel further during deceleration, reducing the PLAs compared to other impact locations.^50^

Composite PLAs and PRAs were seen to decrease consistently as the impact surface angle increased. When impacting an angled surface, the headform undergoes a larger amount of rotation around the axis of the neck compared to a flat impact surface. This causes the headform to roll and slide down the impact surface compared to a flat impact surface, where there is little vertical travel during the deceleration. This effectively increases the distance the head travels during deceleration, lengthening deceleration time slightly. This effect is small in terms of the impact duration change from flat to angled, however it is enough to significantly change the PLA and PRA values.

### Limitations and Further Work

This study aimed to investigate linear and rotational acceleration mitigation of various common soft shelled rugby headgear. This was achieved using an impact testing method inspired by similar investigations from sports where head injuries are prevalent and head protection is employed. The methods were adopted in combination with the relevant sections of the World Rugby standard (mostly pertaining to drop heights). Additionally, the present study aimed to further previous work based around linear-only acceleration analysis of rugby headgear. The methods employed in the study had several limitations.

A comparative impact location for SR boss when testing on a flat impact surface could not be found. When attempting to recreate the SR boss position on the flat, the surface was to contact the side of the rear of the head only. The headform easily and consistently contacted the surface on which the MEP pad was mounted during the impact. It was decided this impact location could not be represented on a flat impact surface and was therefore excluded. Secondly, the impact conditions created in this study may not accurately reflect real life, concussive, head impacts. Unfortunately, analysis of rugby head impacts using high accuracy, well validated instrumentation is lacking. As a result, the impacts used in this study may closely represent those seen on the field, however it is not known how close this representation is. Furthermore, the rotational kinematics generated by this methodology were not compared to those generated by previous research, nor were they compared to those from field studies of un-helmeted sports.

Thirdly, only a limited number of headgear were evaluated as well as an even more limited subset of headgear displaying high impact attenuation performance not approved by world rugby. Finally, only the HIC and RIC were used to relate kinematic data to injury metrics. Several other metrics exist which incorporate both linear and rotational kinematics such as the Head Impact Power, Principle Component Score, and the Brain Injury Criteria. Whilst these were not used in the present study, the authors acknowledge that these would be equally useful in quantifying protective performance.

Further testing of headgear performance on the field is required before any definite conclusions can be drawn on their protective performance. This would require accurate back translation of on-field rugby impacts in the lab, which would serve as a baseline which can then be studied with and without the presence of headgear. Additionally, the effect of the fit of the headgear should be investigated with regards to the impact attenuation behaviour, especially rotational attenuation.

## Conclusions

This experimental method sets the stage for far more in-depth analyses of rugby headgear and its behaviour across all relevant impact locations. This study did not intend to recreate the World Rugby standards or any previous literature but used them as a basis from which to extend the investigation of headgear behaviour. The World Rugby standard assesses only the PLA reduction of headgear using a limited number of impact locations impacted only once each. Since testing rotational acceleration behaviour of soft-shelled rugby headgear has either been poorly reported, or not carried out at all to date, this research could lead the next generation of headgear testing to match the next generation of headgear.

The World Rugby standard explicitly states that headgear approved by world rugby is not intended to protect against any form of mild traumatic brain injury or skull fractures. It makes sense therefore, that headgear 1–3 which have received World Rugby approval, do not reduce PLA or HIC to the same extent as headgears 4 and 5. Headgear 4 has received World Rugby approval, however not through the same standards that headgears 1–3 have. Headgear 4 has been approved through the newer Law 4 assessment trial, for headgear designed to achieve specific, quantifiable medical benefits.

Soft-shelled rugby headgear has demonstrated a potential to reduce both linear and rotational impact accelerations in a laboratory setting. The results of this study show that the headgear tested can lower the PLA by up to 50%, and the PRA by up to 60%. If the accelerations seen in an impact can be lowered to safer levels, the concussive injury risk could potentially be reduced as well. It is not well understood what exactly causes concussion, although the link between high intensity head impacts and concussions is recognised.

## Appendix

See Figs. 9, 10, 11, 12, 13, 14, and 15.
